# Cytokeratin-19 mRNA-positive circulating tumor cells during follow-up of patients with operable breast cancer: prognostic relevance for late relapse

**DOI:** 10.1186/bcr2897

**Published:** 2011-06-10

**Authors:** Emmanouil Saloustros, Maria Perraki, Stella Apostolaki, Galatea Kallergi, Alexandros Xyrafas, Kostas Kalbakis, Sophia Agelaki, Antonia Kalykaki, Vassilis Georgoulias, Dimitris Mavroudis

**Affiliations:** 1 Department of Medical Oncology, University General Hospital of Heraklion, Voutes, Heraklion, 71110, Crete, Greece; 2 Laboratory of Tumor Cell Biology, University of Crete, School of Medicine, Voutes, Heraklion, 71003, Crete, Greece

## Abstract

**Background:**

The detection of cytokeratin-19 (CK-19) mRNA-positive circulating tumor cells (CTC) before and/or after adjuvant chemotherapy in patients with operable breast cancer is associated with poor clinical outcome. Reliable prognostic markers for late disease relapse are not available. In this study we investigated the value of CTC detection during the first five years of follow-up in predicting late disease relapse.

**Methods:**

Blood was analyzed from 312 women with operable breast cancer who had not experienced disease relapse during the first two years of follow-up. A real-time reverse transcriptase polymerase chain reaction (RT-PCR) for CK-19 mRNA was used to detect CTC three months after the completion of adjuvant chemotherapy and every six months thereafter for a follow-up period of five years.

**Results:**

Eighty patients (25.6% of the study population) remained CTC free throughout the five-year period. A change in CTC status was observed in 133 patients (42.6%); 64 patients (20.5%) with initially CK-19 mRNA-positive CTC during the first 24 months turned CTC-negative afterwards while 69 (22.1%) who were initially CTC-negative became CTC-positive. Ninety-nine patients (31.7%) remained persistently CK-19 mRNA-positive. After a median follow-up period of 107 months (range: 38 to 161 months), the persistently CTC-positive patients with either hormonal receptor positive or negative tumors, had a higher risk of late-disease relapse compared to the persistently CTC-negative patients (36.4% versus 11.2%, *P <*0.001). Multivariate analysis revealed that persistently CTC-positive patients also had a shorter disease-free (*P *= 0.001) and overall survival (*P *= 0.001).

**Conclusions:**

Persistent detection of CK-19 mRNA-positive CTC during the first five years of follow-up is associated with an increased risk of late relapse and death in patients with operable breast cancer and indicates the presence of chemo-and hormonotherapy-resistant residual disease. This prognostic evaluation may be useful when deciding on subsequent adjuvant systemic therapy.

## Introduction

Invasive breast cancer is the most common malignancy in women, accounting for 28 percent of new cancer cases and 15 percent of cancer deaths [1]. Due to declining mortality rates that are attributable mostly to the use of screening mammography and effective adjuvant therapy, more women nowadays survive their breast cancer [2]. Since metastatic disease is considered incurable, the early recognition and treatment of potentially still curable minimal residual disease is one of the major goals of care of breast cancer survivors and requires the in-depth understanding of relapse patterns.

Depending on the specific breast cancer type, the majority of recurrences occur during years 2 to 5 [3], although they can occur earlier or much later [4,5]. Especially for women with hormone receptor-positive disease, more than one-half of all recurrences and deaths occur beyond five years from diagnosis [5,6]. To date no tool is available for monitoring the effect of adjuvant treatment and in most cases the recurrence risk is calculated based on previous statistical analyses [7]. Therefore, with existing methods, prediction of the risk of relapse for the individual patient is limited.

Disseminated tumor cells (DTC) in bone marrow [8,9] and circulating tumor cells (CTC) in peripheral blood [10,11] of patients with operable breast cancer have been shown to be independent adverse prognostic factors for disease recurrence and disease-related death. Immunocytochemistry using antibodies against proteins that are expressed on epithelial but not on mesenchymal cells is widely used for the detection of DTC and CTC. However, the detection of mRNA transcripts for specific epithelial markers by using reverse transcriptase polymerase chain reaction (RT-PCR) and, more recently, the quantitative real-time RT-PCR (QPCR) seems to have higher diagnostic sensitivity [12]. The major advantage of RNA-based approaches is related to the rapid degradation of RNA released from cells in the blood by RNAses; therefore, the origin of detectable blood RNA transcripts is considered to be viable cells. Cytokeratin-19 (CK-19), a cytoskeletal component present in normal and cancerous epithelial cells, has been extensively used for the detection of breast cancer cells in mesenchymal tissues and seems to be the most sensitive and reliable tumor marker in both patients with operable and metastatic breast cancer [13,14].

Several studies have shown the prognostic significance of CK-19 mRNA-positive CTC in patients with operable breast cancer [10,11,15-17]. However, all these studies have investigated the prognostic value of CTC at the time of initial diagnosis and before the initiation and/or following the completion of adjuvant chemotherapy. Only a few reports exist concerning the clinical relevance of DTC, but none for CTC, during the surveillance period after the completion of adjuvant chemotherapy [18,19]. The unfavorable clinical outcome of patients with detectable isolated tumor cells in bone marrow was shown in the latter studies [18,19]. Given that DTC and CTC are theoretically the primary targets of adjuvant treatment, their fate after systemic therapy could be a potential useful marker permitting a direct and individualized assessment of treatment efficacy and a more accurate estimation of the risk of relapse.

In the present study, we sought to evaluate the clinical relevance of CK-19 mRNA-positive CTC detected by a QRT-PCR assay at different time points during the follow up period after the completion of adjuvant chemotherapy in patients with operable breast cancer. We hypothesized that patients presenting detectable CK-19 mRNA-positive CTC during follow-up despite the administration of adjuvant therapy, could be at an increased risk of late disease relapse (defined as relapse at least two years after the end of adjuvant chemotherapy) and death.

## Materials and methods

### Patients and clinical samples

We conducted a retrospective analysis of prospectively collected data in the context of an ongoing longitudinal study that has been previously reported [17]. Women with operable breast cancer (stage I to III) who were under surveillance and had not experienced disease relapse during the first two years of follow-up, were eligible for this study. All patients had received adjuvant chemotherapy mostly in the context of research protocols of the Hellenic Oncology Research Group. After completion of adjuvant chemotherapy, patients received adjuvant radiotherapy and hormonal therapy when indicated according to their individual disease characteristics. There were no subgroups of patients who received only adjuvant hormone therapy or no adjuvant systemic therapy at all.

Patients' follow-up consisted of pertinent medical history and physical examination, with laboratory and imaging studies restricted as indicated, every three months for the first two years, every six months for the next three years and yearly thereafter. All treating physicians were completely unaware of the CK-19 mRNA results for their individual patients and all follow-up laboratory and imaging studies to detect disease relapse were performed independently of the CK-19 mRNA results. All patients signed an informed consent to participate in the study, which was approved by the Ethics and Scientific Committees of our institution.

Cytokeratin-19 mRNA-positive CTC were monitored at specific time points after the completion of adjuvant chemotherapy for a five-year follow-up period. The first blood sample was obtained three months after the end of chemotherapy and subsequent samples were obtained every six months thereafter during the five-year follow-up.

Patients were classified into four groups based on their CTC status during the first two years and the subsequent three years of follow-up (as persistently negative, persistently-positive, negative turn to positive and the opposite). At least one CK-19 mRNA-positive blood sample in the corresponding period of time was required for classifying the patient in the CTC-positive group. On the other hand, if all the collected blood samples were negative for CK-19 mRNA, the patient was characterized as CTC-negative. Using this definition, the patients were classified in the "persistently CTC-negative" group, if there were no positive blood samples for CK-19 mRNA throughout the five-year follow-up period. On the other hand, the "persistently CTC-positive" patients had at least one positive blood sample for CK-19 mRNA in the first two years and at least another positive one in the subsequent three years of follow-up. Accordingly, patients in the "CTC-negative turn to positive" group had no positive samples in the first two years, but at least one positive sample in the next three years. The opposite was true for the "CTC-positive turn to negative" group.

### Blood samples and real-time RT-PCR for CK-19 mRNA

Twenty milliliters (mL) of peripheral blood in EDTA were collected at each visit. To avoid contamination with epithelial skin cells, all blood samples were obtained at the middle of vein puncture after the first 5 mL of blood was discarded. Peripheral blood mononuclear cells were obtained by gradient density centrifugation using Ficoll-Hypaque [10]. Total RNA isolation was carried out with the use of Trizol LS reagent (Gibco, Life Sciences, BRL, Grand Island, NY, USA) according to the manufacturer's instructions. The isolated RNA was dissolved in diethylpyrocarbonate-treated water and stored at -80°C until used. RNA concentration was determined by absorbance reading at 260 nm with the Hitachi UV-VIS (U-2000) spectophotometer (Tokyo, Japan). The integrity was tested by PCR amplification of the β-actin housekeeping gene. As positive and negative controls we used RNA samples prepared from the MCF-7 breast cancer and ARH-77 leukemic cell lines respectively.

Reverse transcription of RNA was carried out with the Thermoscript RT-PCR system (Invitrogen, Paisley, UK). Complementary DNA (cDNA) was synthesized according to the manufacturer's instructions. The QPCR assays for the detection of CK-19 mRNA-positive cells, the primers and the details of the cycling protocol have been previously described [20]. Briefly, 2 μl of cDNA were placed into an 8 μl reaction volume containing 1 μl of the sense primer CK-19-for (3 mM), 1 μl of the antisense primer CK-19-do (3 mM), 2.4 μl of the LightCycler Fast Start DNA Master Hybridization Probes reagent (10 × concentration), 1 μl of the probe CK-19-FL (3 mM) and 1 μl of the probe CK-19-LC (3 mM) [15]. The quality of cDNAs was evaluated by real-time PCR for the housekeeping gene glyceraldehyde-3-phosphate dehydrogenase.

The presence of more than 0.6 MCF-7 cell equivalents/5 μg of total RNA was considered a positive result, according to the previously reported analytic detection limit of our assay [20]. Using this cutoff, only 2 of 89 (2.2%) female healthy blood donors were positive for CK-19 mRNA-positive cells while none of 9 women with benign breast disease (fibroadenomas) had positive blood samples [20]. The high specificity of the method was made possible by avoiding contamination of skin epithelial cells during venipuncture, as well as by carefully designing the primers and hybridization probes. Hence, amplification of the known CK-19 pseudogenes and genomic DNA was avoided [20].

### Statistical analysis

Disease-free survival (DFS), defined as the time from study entry until the day of the first evidence of disease recurrence, and overall survival (OS), defined as the time from study entry to death, were the main dependent variables of the study. The data-cut-off date was 20 July 2010. Kaplan-Meier curves for DFS and OS were compared using the log-rank test to provide a univariate assessment of the prognostic value of selected clinical risk factors. Clinicopathologic factors known to be associated with prognosis, such as menopausal status (premenopausal *vs *postmenopausal), tumor size (T1 *vs *T2-3), number of the involved axillary lymph nodes (0 to 3 *vs *≥ 4), histological grade (1 or 2 *vs *3), estrogen receptor (ER) status (negative *vs *positive), progesterone receptor (PR) status (negative *vs *positive) and HER-2/*neu *status (negative *vs *positive) were tested in univariate analysis. Variables that were found to be significant at the univariate screen were then entered in a stepwise multivariate Cox proportional hazards regression model to identify those with independent prognostic information. Entry into and removal from the model were set at 5% and 10%, respectively. All statistical tests were performed at the 5% level of significance. SPSS version 13 (SPSS Inc, Chicago, IL, USA) statistical software was used for the analysis. This report is written according to the reporting recommendations for tumor marker prognostic studies (REMARK criteria) [21].

## Results

### Patients' characteristics

A total of 455 consecutive patients with diagnosis of operable breast cancer treated and followed at the Department of Medical Oncology of the University Hospital of Heraklion between January 1997 and December 2004 were screened for eligibility for this study. A total of 412 (91%) patients belong to the same cohort that was used to evaluate the prognostic significance of CK-19 mRNA-positive CTC detection before initiation and/or after completion of adjuvant chemotherapy [17].

**Figure 1:**
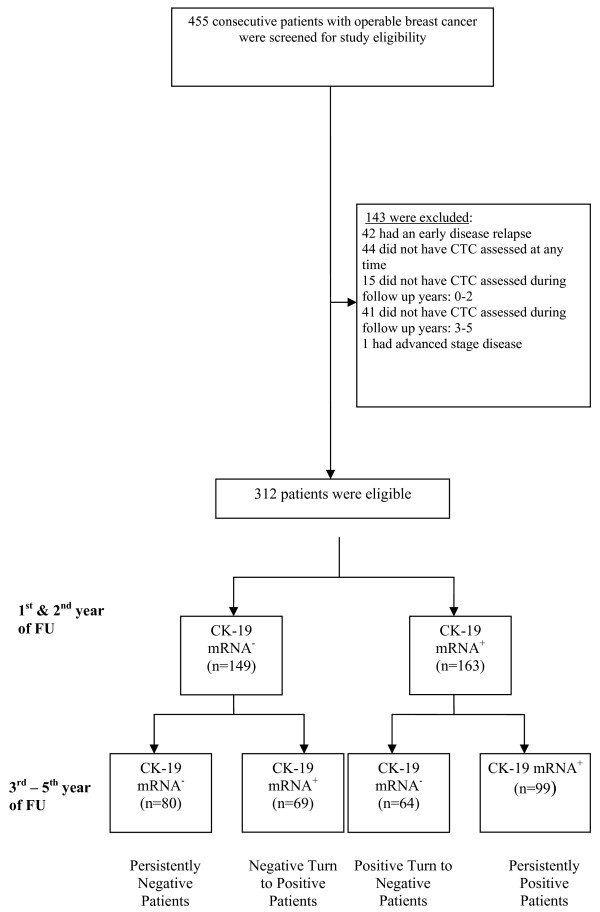
**Study enrollment, reasons for patients' exclusion and patients' classification according to CK-19 mRNA CTC status**.

A total of 143 patients were excluded for reasons listed in Figure 1 and 312 were included in the study. Patients' characteristics at the time of primary diagnosis in relation to CTCs status during follow-up are summarized in Table 1. The persistent detection of CTC during follow-up did not correlate with the patient's and/or tumor's characteristics, such as age (*p *= 0.197), menopausal status (*p *= 0.372), tumor size (*p *= 0.637), lymph node status (*p *= 0.082), histopathological grade (*p *= 0.746) and hormone receptor status (*p *= 0.156). There was a difference in the type of adjuvant chemotherapy administered, with more patients in the persistently positive group having received anthracycline-based regimens (*p *= 0.011).

**Table 1:** Patients' characteristics at diagnosis according to CK-19 mRNA-positive cells during follow-up

Characteristics	All Patients		CK-19 mRNA-persistently negative		CK-19 mRNA-persistently positive		
	N	%	N	%	N	%	
Patients enrolled	312		80	25.6	99	31.7	
Age							Mann-Whitney *P *= 0.197
median (range)	54 (26 to 77)		51.5 (26 to 75)		54 (28 to 75)		
Menopausal status							*P *= 0.372
Pre-menopausal	145	46.5	41	51.3	44	44.4	
Post-menopausal	167	53.5	39	48.8	55	55.6	
Tumor size							T2/T3 vs T1
T1	116	37.2	30	37.5	33	33.3	*P *= 0.637
T2	174	55.8	43	53.8	59	59.6	
T3	22	7.1	7	8.8	7	7.1	
Lymph nodes							*P *= 0.082
N0	107	34.3	33	41.3	28	28.3	
N1 to 3	119	38.1	29	36.3	35	35.4	
N > 3	86	27.6	18	22.5	36	36.4	
Histology grade							*P *= 0.746
1/2	159	51.0	39	48.8	56	56.6	
3	119	38.1	28	35.0	36	36.4	
lobular	34	10.9	13	16.3	7	7.1	
HR							*P *= 0.156
ER(-)/PR(-)	70	22.4	24	30.0	22	22.2	
Other	190	60.9	39	48.8	60	60.6	
Unknown	52	16.7	17	21.3	17	17.2	
HR and Her-2							*P *= 0.116
ER(-)/PR(-)/HER-2(-)	52	16.7	19	23.8	16	16.2	
Other	255	65.1	41	51.3	66	66.7	
Unknowm	57	18.3	20	25.0	17	17.2	
Radiation therapy							*P *= 0.937
No	24	7.7	7	8.8	9	9.1	
Yes	289	92.3	73	91.2	90	90.9	
Hormonotherapy							*P *= 0.103
No	23	7.4	10	12.5	5	5.1	
Yes	289	92.6	70	87.5	94	94.9	
Type of hormonotherapy							*P *= 0.119
No hormonotherapy	23	7.4	10	12.5	5	5.1	
AIs	33	10.6	10	12.5	11	11.1	
T	50	16.0	10	12.5	19	19.2	
AIs & T	57	18.3	9	11.3	22	22.2	
LHRH	32	10.3	11	13.8	8	8.1	
LHRH + T or AIs	117	37.5	30	37.5	34	34.3	
Chemotherapy							*P *= 0.011
CMF	30	9.6	11	13.8	4	4.0	
FEC	149	47.8	31	38.8	57	57.6	
T/EC	133	42.6	38	47.5	38	38.4	

AIs, aromatase inhibitors; CMF, cyclophosphamide-methotraxate-fluorouracil; FEC, fluorouracil-epirubicin-cyclophosphamide; HR, hormone receptor; LHRH, luteinizing-hormone-releasing hormone; T, tamoxifen; T/EC, taxane/epirubicin-cyclophosphamide.

### Detection of CK-19 mRNA-positive cells during follow-up

Cytokeratin-19 mRNA-positive cells were detected in the blood of 232 patients (74.4%) at any time point during the five-year follow-up period, while 80 patients (25.6%) remained CTC-free throughout the same period (persistently negative). More specifically, 99 patients (31.7%) had persistently detectable CK-19 mRNA-positive cells both during the first two and the subsequent three years of follow-up (persistently-positive group). A change in CK-19 mRNA status was observed in almost half of patients (133 patients or 42.6%). Of those, 64 patients (20.5%) with initially detectable CK-19 mRNA-positive cells during the first 24 months turned CTC-negative afterwards (positive turn to negative group), while 69 patients (22.1%) who were initially CTC-negative became CTC-positive afterwards (negative turn to positive group) (Figure 1).

### Detection of CK-19 mRNA-positive cells and clinical outcome

#### Disease recurrence

After a median follow-up period of 107 months (range: 38 to 161 months), 63 patients (20.2%) had developed a distant (*n *= 56; 88.8%) or locoregional disease recurrence (*n *= 7; 11.2%) (Table 2). Compared to the persistently negative patients, only the group of CK-19 mRNA-persistently positive patients had a significant higher risk of disease relapse (36.4% versus 11.2%; Fisher's exact test, *p *< 0.001). In fact, risk of disease recurrence was the highest in patients with persistently-positive CTC (36.4% versus 7.8%; *p *< 0.001 and 36.4% versus 18.8%; *p *= 0.016 compared to a positive turn to negative and a negative turn to positive group, respectively) (Table 2).

**Table 2:** Incidence of disease recurrence and deaths according to the detection of CK-19 mRNA-positive circulating tumor cells

CK-19 mRNA	No of patients	Relapses			Deaths		
		Yes N (%)	No N (%)	Fisher's Exact test, *P*	Dead N (%)	Alive N (%)	Fisher's Exact test, *P*
**Persistently Positive**	99	36 (36.4)	63 (63.6)	*P *< 0.001	24 (24.2)	75 (75.8)	*P *= 0.001
**Persistently Negative**	80	9 (11.2)	71 (88.8)		5(6.3)	75 (93.8)	
**Positive Turn to negative**	64	5 (7.8)	59 (92.2)	*P *< 0.001 versus persistently positive	3 (4.7)	61 (95.3)	*P *= 0.001 versus persistently positive
**Negative Turn to positive**	69	13 (18.8)	56 (81.2)	*P *= 0.016 versus persistently positive	9 (13.0)	60 (87.0)	*P *= 0.079 versus persistently positive

While also,

*P *= .248 for relapse of turn to positive versus persistently negative

*P *= .172 for deaths of turn to positive versus persistently negative

CK-19, cytokeratine-19.

**Figure 2:**
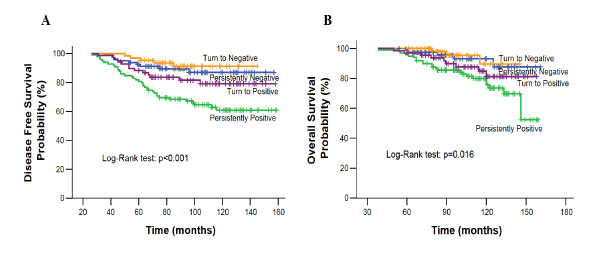
**(A): Disease-free and (B): Overall Survival according to CTC detection during follow-up**.

The five-year DFS rates were 82.5% versus 92.7% for persistently-positive versus persistently-negative patients, respectively. As illustrated in Figure 2A, persistently positive patients had a significantly shorter DFS than the persistently negative patients (*p *< 0.001). Although no group has as yet reached the median DFS, there was a progressive decrease in the DFS of the four groups of patients according to the detection of CK-19 mRNA-positive CTC during the five years of follow-up (Figure 2A).

#### Survival

Forty-one patients (13.1%) died during follow-up as a result of disease progression. Twenty-four (58.5%) and five (12.2%) of these deaths occurred in the persistently-positive and persistently-negative group, respectively (Fisher's exact test; *p *= 0.001; Table 2). The 10-year overall survival rates were 81.4% for persistently-positive versus 96.7% for persistently-negative patients. Estimated median overall survival was significantly shorter for persistently positive compared to persistently negative patients (*p *= 0.013). Similar to DFS, there was a progressive decrease in the OS rates of the four groups of patients according to the detection of CK-19 mRNA-positive CTC during the five years of follow up (Figure 2B).

### Subgroup analysis based on cumulative number of positive tests for pre-chemotherapy CTC status and hormone receptors

Since patients underwent serial blood draws for the assessment of CK-19 mRNA-positive CTC, we analyzed our data to address the question whether the cumulative number of positive tests matters. Among patients with positive tests, 38.7% (during the first two years of follow-up), 24.4% (during the subsequent three years) and 57.3% (during all the five years) had two or more positive test results (Additional file 1, Supplementary Table S1). No difference was found in the disease-free survival between the groups with different cumulative number of positive tests, probably due to the small number of patients and events in each group (Additional file 1: Supplementary Figure S1).

Given the prognostic role of the CTC detection before the administration of adjuvant chemotherapy [11], we investigated whether it could offer additional prognostic information to that of the serial measurements of CTC during follow-up. For this purpose, we reviewed the pre-chemotherapy CTC status of the patients included in this analysis (Additional file 1: Supplementary Table S2). No difference was found in the detection rate of the CK-19 mRNA-positive CTC between the four groups (Pearson chi-square, *p *= 0.320). Interestingly, the persistently positive patients with detectable CK-19 mRNA-positive CTC before the administration of adjuvant chemotherapy had shorter DFS but not OS compared to the patients of the same group who tested negative for pre-chemotherapy CK-19 mRNA-positive CTC (Additional file 1: Supplementary Figure S2).

**Figure 3:**
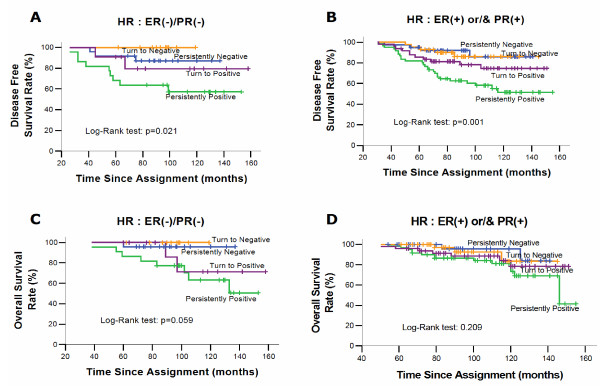
**Disease-free and overall survival according to the CTC detection and hormone receptor status during follwow-up**.

Finally, a subgroup analysis was performed according to hormone receptor status. Interestingly, the persistently positive patients with either hormone receptor positive or negative tumors had a significantly higher relapse rate (Table 3), risk of death and shorter DFS than the persistently negative patients [(*p *= 0.039 and *p *= 0.004 for persistently positive vs persistently negative patients with ER/PR negative and ER and/or PR positive tumors respectively) (Figure 3a, 3b)]. However, the overall survival was shorter only for the persistently positive patients with ER/PR negative tumors (*p *= 0.035, Figures 3c, 3d).

**Table 3:** Incidence of Disease recurrence and deaths according to CTC detection and hormonal receptor status

HR Status	CK-19 mRNA	No of patients	Relapses			Deaths		
			Yes N (%)	No N (%)	Fisher's Exact test, *P*	Dead N (%)	Alive N (%)	Fisher's Exact test, *P*
**ER(-)/PR(-)**	**Persistently Positive**	22	9 (40.1)	13(59.9)	*P *= 0.044	8 (34.4)	16 (63.6)	*P *= 0.009
	**Persistently Negative**	24	3 (12.5)	21 (87.5)		1(4.1)	24 (95.9)	
**ER(+) and/or PR(+)**	**Persistently Positive**	60	26 (43.3)	34 (46.7)	*P *< 0.001	15 (25)	45 (75)	*P *= 0.007
	**Persistently Negative**	39	4 (10.2)	35 (89.2)		2 (5.1)	37 (94.9)	

CK-19, cytokeratine-19; HR, hormonal receptor; ER, estrogens receptor; PR, progesterone receptor.

### Univariate and multivariate analysis

Persistent detection of CK-19 mRNA-positive CTC during the follow-up after the completion of adjuvant chemotherapy, tumor size greater than 2.0 cm, more than three involved axillary lymph nodes and postmenopausal status were significantly associated with reduced DFS and OS in the univariate analysis (Table 4). Multivariate analysis revealed that persistent detection of CK-19 mRNA-positive CTC, tumor size and more than three involved axillary lymph nodes were independent prognostic factors for shorter DFS and OS (Table 5).

**Table 4:** Univariate analysis (unadjusted relative risks) for disease-free and overall survival

	DFS			Overall survival		
	**HR**	**95% CI**	***P*-value**	**HR**	**95% CI**	***P*-value**

**Menopausal status**		1.075 to 3.064			1.027 to 3.836	
Post vs Pre	1.815		**0.026**	1.985		**0.041**
**Tumor status**		1.304 to 4.419			1.710 to 11.114	
T2/T3 vs T1	2.401		**0.005**	4.359		**0.002**
**Nodes**		1.418 to 3.832			1.527 to 5.207	
N> 3 vs N0-3	2.331		**0.001**	2.819		**0.001**
**Histology grade**		0.989 to 2.805			0.644 to 2.390	
3 vs 1/2	1.665		0.055	1.240		0.520
**HR**		0.454 to 1.504			0.585 to 2.387	
ER(-)/PR(-) vs other	0.826		0.533	1.182		0.641
**Triple negative**		0.454 to 1.681			0.492 to 2.348	
ER(-)/PR(-)/HER-2 vs Other	0.874		0.686	1.075		0.856
**Hormonotherapy**		0.696 to 3.759			0.442 to 4.687	
No vs Yes	1.618		0.264	1.439		0.546
**Chemotherapy**	CMF (ref)		0.589 to 3.812			0.352 to 2.976
FEC			0.339			0.911
T/EC	1.498	0.389 to 2.745	0.396	1.024	0.388 to 3.564	0.966
	1.034		0.947	1.176		0.775
**CK-19 at five years FU** Persistently negative Turn to negative Turn to positive Persistently positive (ref)	0.300 0.201 0.498	0.144 to 0.623 0.079 to 0.513 0.264 to 0.939	0.001 0.001 0.031 **< 0.001**	0.318 0.260 0.582	0.121 to 0.836 0.078 to 0.868 0.271 to 1.253	0.020 0.028 0.167 **0.025**

CK-19, cytokeratine-19; CMF, Cyclophosphamide-Methotraxate-Fluorouracil; DFS, disease-free survival; FEC, Fluorouracil-Epirubicin-Cyclophosphamide; HR, hormone receptor; T/EC, Taxane/Epirubicin-Cyclophosphamide.

**Table 5:** Prognostic factors by multivariate analysis for disease-free and overall survival

	DFS			Overall survival		
	**HR**	**95% CI**	***P*-value**	**HR**	**95% CI**	***P*-value**

**Menopausal status** Post vs Pre	1.462	0.858 to 2.492	**0.163**	1.365	0.794 to 2.347	**0.260**
**Tumor status** T2/T3 vs T1	2.187	1.167 to 4.098	**0.015**	2.135	1.141 to 3.994	**0.018**
**Nodes** N> 3 vs N0-3	1.801	1.080 to 3.003	**0.024**	2.150	1.109 to 4.168	**0.023**
**CK-19 at five years FU** Persistently negative Turn to negative Turn to positive Persistently positive (ref)	0.328 0.206 0.622	0.157 to 0.683 0.081 to 0.526 0.327 to 1.186	0.003 0.001 0.149 **0.001**	0.330 0.201 0.587	0.159 to 0.688 0.079 to 0.514 0.309 to 1.115	0.003 0.001 0.104 **0.001**

CK-19, cytokeratine-19; CMF, cyclophosphamide-methotraxate-fluorouracil; DFS, disease-free survival; ER, estrogen receptor; FEC, fluorouracil-epirubicin-cyclophosphamide; PR, progesteron receptor; T/EC, taxane/epirubicin-cyclophosphamide.

## Discussion

We provide, to our knowledge, the first clear evidence of a strong correlation between detection of CK-19 mRNA-positive CTC during follow-up and increased risk of late disease relapse and death in patients with either hormonal receptor positive or negative operable breast cancer. These findings support the role of CTC monitoring as an adjunct to standard clinical and radiographic methods in the evaluation of disease status during follow-up.

Although the prognostic role of DTC for disease relapse and death in early breast cancer is clearly documented [8], the assessment of tumor cells in peripheral blood is easier, more broadly applicable than bone marrow aspirates and certainly more appropriate for repeated testing. Our group has previously reported that the detection of CK-19 mRNA-positive CTC in the blood of patients with node-negative operable breast cancer before the initiation of any systemic treatment was an independent prognostic factor associated with an increased risk of disease recurrence [11]. More recently, we demonstrated that the risk of relapse could be distinguished based on the response of their CTC to adjuvant chemotherapy [17] and we reviewed our experience regarding the prognostic role of CK-19 positive CTC in operable breast cancer [22]. Other investigators have shown that longitudinal monitoring of CTC was superior to a single test analysis, and a more than 10-fold increase in the CTC numbers towards the end of therapy is highly predictive for early relapse [23].

The present study was designed to investigate the prognostic value of CTC detection during follow-up in predicting the risk of late disease relapse. Accordingly, patients who experienced disease relapse during the first two years from diagnosis were excluded from this analysis and the median follow-up period was extended to 107 months. The changes in CK-19 mRNA-positive CTC status were thus analyzed in 312 patients and four groups were distinguished. The first group included patients without detectable CTC throughout the follow-up period, only 11.2% of whom experienced disease relapse. The second group included patients with CK-19 mRNA-positive CTC during the first two years. These patients had similar relapse risk to the persistently negative patients and might indeed have derived a benefit from the adjuvant therapy. In the third group patients with detectable CTC after, but not during, the first two years were included. The relapse risk for these patients was almost 50% higher compared to the risk of persistently negative patients, presumably due to the growth of therapy-resistant residual disease, which could not be detected early on by our method. Finally, in the fourth group patients with CK-19 mRNA-positive CTC throughout the follow-up period were included. One third of them experienced disease relapse, while one out of four patients died. This persistently positive group was by far the group with the highest relapse risk.

Almost 40% of the "positive at any time" patients had detectable CK-19 mRNA-positive CTC only in one single sample. This observation could theoretically be attributed at least in part to false positive results. However, given the very low false positive rate of our assay (approximately 2%) this occurrence should be rather limited.

Our results could be explained by the hypothesis that drug-resistant cancer cell clones generated during tumor evolution, constitute the re-emerging dominant tumor cell population and may start proliferating under the selective pressure of drug exposure. The high probability of subsequent disease relapse indicates that these resistant cells have a proliferative and survival advantage. This hypothesis seems to be supported by the observation (unpublished data) that the vast majority of CTC detected during the follow-up period from CK-19 mRNA persistently positive patients who had experienced disease relapse, did not express the M30 antigen which is a neo-epitope expressed only after caspase cleavage of cytokeratin 18 during early apoptosis [24,25].

An algorithm for the optimal timing of CTC detection during follow-up is currently lacking. Our findings suggest that serial assessments every six months for up to five years represent an acceptable timetable. Furthermore, for hormone receptor positive patients, in whom half of the recurrences occur beyond the five years, extension of the CTC serial assessments for even longer might be reasonable. These patients may represent the group which could derive benefit from an extended adjuvant treatment approach or switch to another agent.

The QRT-PCR used in our study is not the only available assay for CTCs' detection. A semi-automated approach, the CellSearch^® ^system (Veridex Raritan, NJ, USA), which has been approved by the Food and Drug Administration for monitoring CTC in the metastatic breast cancer setting, has gained considerable attention [26,27]. The prognostic relevance of CTC detection in the peripheral blood of operable breast cancer patients, using the CellSearch^® ^system, has been evaluated in the SUCCESS trial. According to the most updated results, detection of at least one CTC in 23 mL of peripheral blood after surgical resection of the primary tumor and before the start of adjuvant systemic treatment was an independent predictor for worse DFS and OS in multivariate analysis [28]. The prognostic significance of CTC detection before and/or after the completion of adjuvant chemotherapy [17], as well as the high specificity/sensitivity represent some advantages of the QPCR assay compared to the CellSearch^®^. On the other hand, the ability for direct enumeration, morphological analysis and isolation of CTC for further analysis are important advantages of the latter [29].

Our study has some potential limitations that should be taken into account when considering the results. This is a single institution study and the analysis was performed in one laboratory. Therefore, before the establishment of our assay as a clinically relevant test, sample analysis must be performed in several laboratories and stability during shipment must be demonstrated. Also and despite the fact that our assay has been validated in multiple cohorts and data analyses [10,11,14,15,17], neither survival advantage, nor improvement in quality of life has been demonstrated in a prospective randomized trial. In this regard, the SWOG and the Breast Cancer Intergroup of North America have initiated a prospective trial in the metastatic setting to test whether patients with elevated CTC count (using the CellSearch^® ^system) after one cycle of first-line chemotherapy will benefit from a switch to a different chemotherapeutic regimen (SWOG protocol S0500). However, for patients with operable breast cancer the lower CTC detection rate post-chemotherapy makes this strategy far more challenging [28,30].

Additionally, the patients in our study received various types of adjuvant therapy based on available clinical and disease data at the time of enrolment. This heterogeneity may be a confounding variable, but the similarity between our findings on relapses (20.2%) and those published by the Early Breast Cancer Trialists' Collaborative Group is encouraging [6]. Finally, the cellular heterogeneity of CTCs was not analysed using the QRT-PCR detection method. This is very important since various studies have already confirmed that CTC present significant genetic and phenotypic heterogeneity [31], which could explain why not all patients who have detectable CTC experience disease relapse, while some patients relapse although they do not present detectable CTC.

## Conclusions

Our data support a prognostic role and potential clinical utility of monitoring CTC in conjunction with standard surveillance strategies for the follow-up of patients with operable breast cancer. Given their independent unfavorable prognostic value for reduced DFS and OS, the detection of CTC after therapy could be considered as indirect evidence for the presence of chemotherapy and hormonal therapy resistant disease. Analyzing a 20-mL blood sample at various time points during follow-up might, therefore, enable clinicians to assess the efficacy of administered adjuvant therapy, limit patient exposure to ineffective agents with unnecessary toxicity, assist in the identification of patients who are most likely to benefit from clinical trials of novel therapeutics and perhaps make eradication of cancer cells more feasible, when the tumor burden is still low and before the appearance of clinically overt metastases. Since only one-third of patients with persistent CK-19 mRNA-positive CTC experience disease relapse, additional prognostic markers are needed to define better those patients who indeed might benefit from novel extended adjuvant therapies. These hypotheses can be addressed only in the context of well-designed, adequately powered, prospective, randomized clinical studies. In this way, definitive proof will be provided as to whether monitoring of the CTC can be used to improve clinical outcome in patients with operable breast cancer.

## Abbreviations

cDNA: complementary deoxyribonucleic acid; CK-19: cytokeratin-19; CTC: circulating tumor cells; DFS: disease-free survival; DNA: deoxyribonucleic acid; DTC: disseminated tumor cells; ER: estrogen receptor; mL: milliliters; mRNA: messenger ribonucleic acid; OS: overall survival; PR: progesterone receptor; RT-PCR: real-time reverse transcription-polymerase chain reaction.

## Competing interests

The authors declare that they have no competing interests.

## Authors' contributions

ES participated in the design of the study, collected the data, analyzed the results and drafted the manuscript. MP, SA and GK: performed the laboratory work. KK, AK and SAg took care of the patients. AX performed the statistical analysis. VG and DM conceived and supervised the study and reviewed the manuscript. All authors have read and approved the final manuscript.

## Supplementary Material

Additional file 1 **Supplemental material**. Word document containing Supplementary Tables S1 and S2 and Supplementary Figures S1 and S2. Click here for file
