# Supplementary material for: Cytokeratin-19 mRNA-positive circulating tumor cells during follow-up of patients with operable breast cancer: prognostic relevance for late relapse
Source: Breast Cancer Res. 2011 Jun 10;13(3):R60. doi: 10.1186/bcr2897 (PMC3218949; doi:10.1186/bcr2897)

| *Number of positive analyses* | *First 2 years of follow up N (%)* | *Subsequent 3 years of follow up N (%)* | *Entire 5 years of follow up N (%)* |
| --- | --- | --- | --- |
| 1 | 100 (61.4) | 121 (72) | 99 (42.6) |
| 2 | 32 (19.6) | 29 (17.3) | 62 (26.8.) |
| 3 | 25 (15.3) | 13 (7.7) | 28 (12.1) |
| ≥4 | 6 (3.7) | 5 (3) | 43 (18.5) |
| Total | 163 | 168 | 232 |

**Supplementary table 1**: Distribution of number of positive samples during the first 2 years, the subsequent 3 years and the entire 5 years of follow up.

**Supplementary figure 1**: Disease-free survival according to the cumulative number of positive analyses during: (A) the first two years, (B) the subsequent three years and (C) the total five years of follow up.


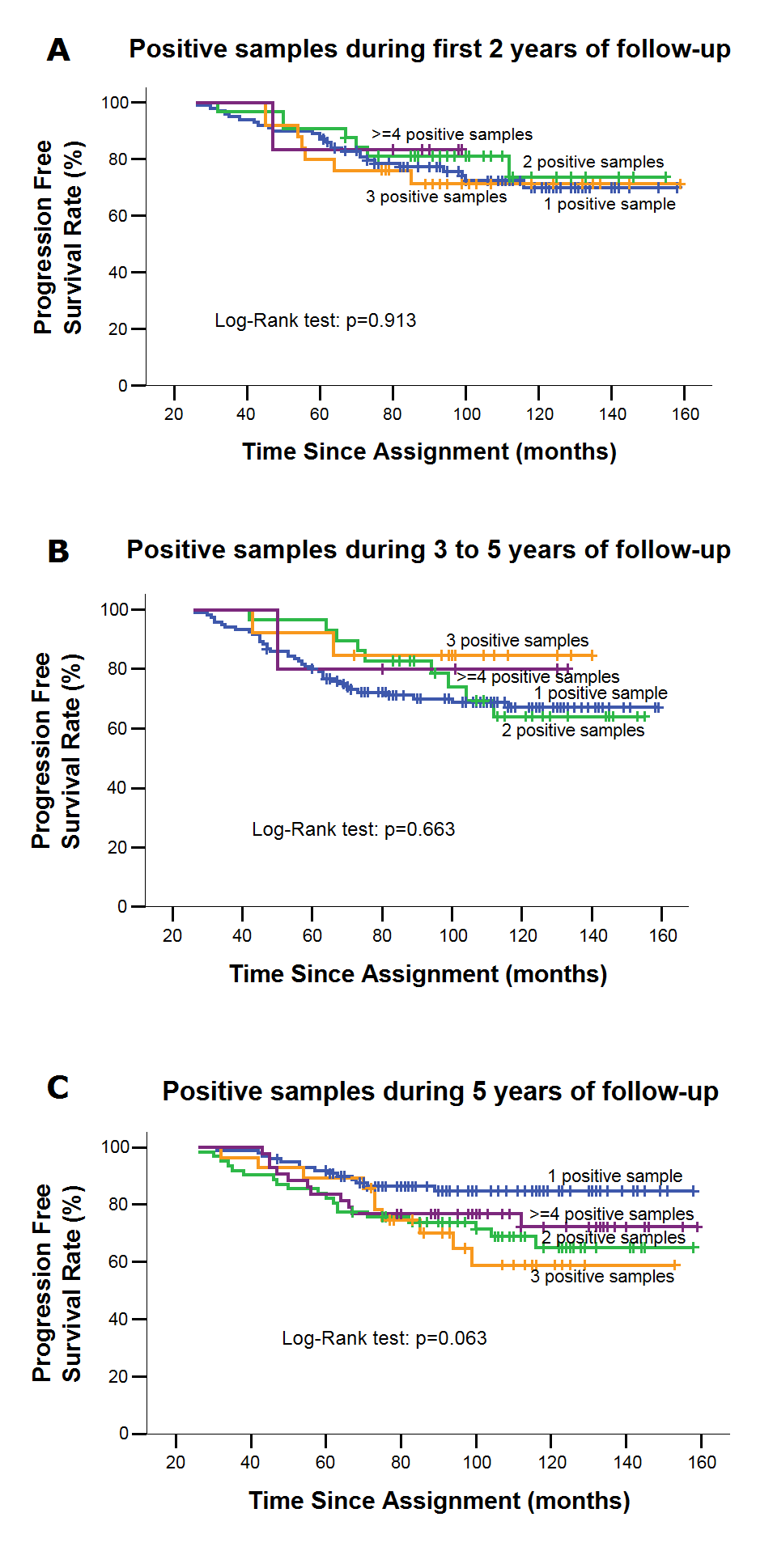


|  | | Disease free survival | | | Overall survival | |
| --- | --- | --- | --- | --- | --- | --- |
| **CK-19 mRNA**  **5 years of follow up** | **CK-19 mRNA**  **Pre-chemotherapy** | **Total** | **Events** | **Rate (%)** | **Events** | **Rate (%)** |
| Persistently Negative | Negative | 47 | 8 | 17 | 4 | 8.5 |
|  | Positive | 32 | 1 | 3.1 | 1 | 3.1 |
| Turn to negative | Negative | 32 | 1 | 3.1 | 1 | 3.1 |
|  | Positive | 31 | 4 | 12.9 | 2 | 6.4 |
| Turn to positive | Negative | 32 | 5 | 13.5 | 3 | 7.9 |
|  | Positive | 31 | 8 | 25.8 | 6 | 19.3 |
| Persistently Positive | Negative | 31 | 11 | 31.4 | 9 | 25.7 |
|  | Positive | 53 | 24 | 45.3 | 15 | 28.3 |

**Supplementary table 2**: Incidence of disease recurrence and deaths according to the pre-chemotherapy detection of CK-19 mRNA-positive circulating tumor cells.

Abbreviations: CK-19, cytokeratine-19.

**Supplementary figure 2**: Disease-free (A) and overall (B) survival of the persistently positive patients according to the pre-chemotherapy CK-19 mRNA positive CTCs’ status.


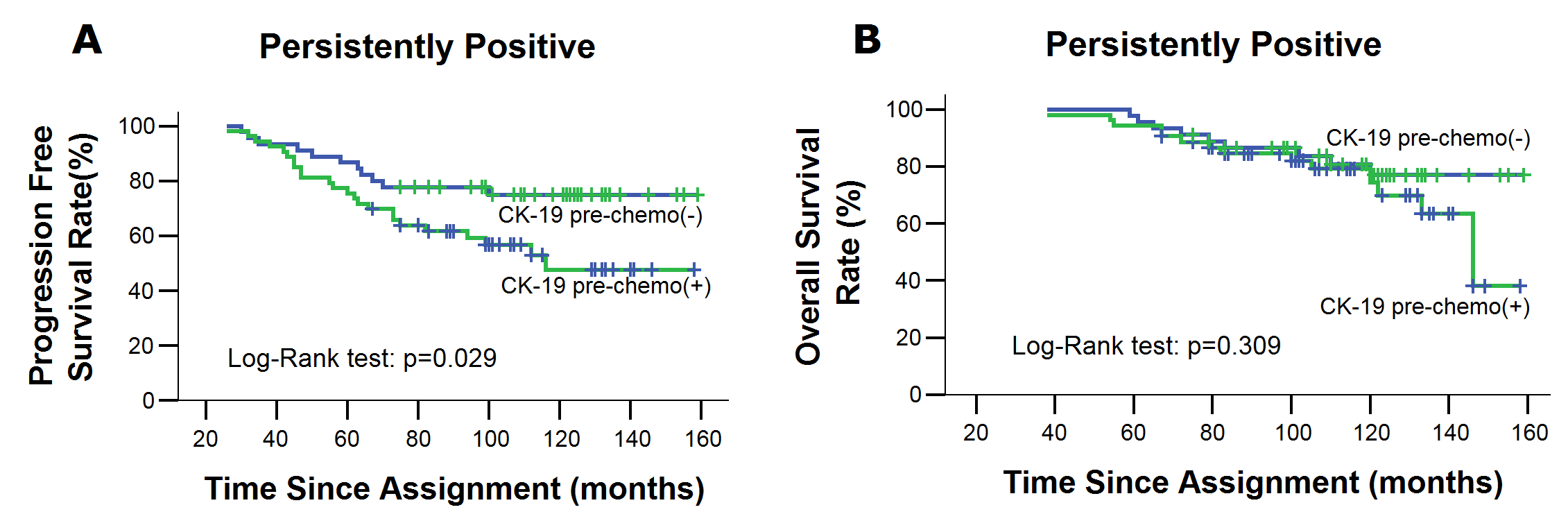

Supplement: Additional file 1 — Supplemental material. Word document containing Supplementary Tables S1 and S2 and Supplementary Figures S1 and S2. [file bcr2897-S1.DOC]
